# New Ultrasonographic Parameter for Diagnosing Carpal Tunnel Syndrome: The Median Nerve-to-Ulnar Artery Cross-Sectional Area Ratio

**DOI:** 10.3390/medicina61112002

**Published:** 2025-11-08

**Authors:** Junhee Lee, So-Youn Chang, Joon Shik Yoon

**Affiliations:** 1 Department of Physical Medicine and Rehabilitation, Hallym University Sacred Heart Hospital, Anyang 14068, Republic of Korea; jhl3728@gmail.com; 2 Department of Physical Medicine and Rehabilitation, Korea University College of Medicine, Seoul 02841, Republic of Korea; 3 Department of Physical Medicine and Rehabilitation, Yeouido St. Mary’s Hospital, College of Medicine, The Catholic University of Korea, Seoul 07345, Republic of Korea; jsy83927@hanmail.net; 4 Department of Physical Medicine and Rehabilitation, Korea University Guro Hospital, Seoul 08308, Republic of Korea

**Keywords:** carpal tunnel syndrome, ultrasonography, median nerve-to-ulnar artery cross-sectional area ratio

## Abstract

*Background and Objectives:* Carpal Tunnel Syndrome (CTS) is the most common entrapment neuropathy. Ultrasonography is a widely used diagnostic method for CTS, and the median-to-ulnar nerve cross-sectional area ratio (MUR) is a well-known parameter. However, the ulnar nerve may be affected by various conditions of the median nerve; therefore, we considered finding further parameters. The aim of the study is to identify the correlation between the median nerve-to-ulnar artery cross-sectional area ratio (MUAR) and existing ultrasonographic parameters used as diagnostic indicators of CTS. *Materials and Methods:* Sixty-seven wrists from forty-two patients who were diagnosed with CTS by electrodiagnostic studies within 4 years before enrollment were retrospectively analyzed in this study. The median nerve cross-sectional area (CSA), ulnar nerve CSA, and ulnar artery CSA at full dilation were measured. In addition to well-defined CTS ultrasonographic parameters, the MUAR were calculated. All measurements were gathered at three levels. The reproducibility was evaluated through intraclass correlation coefficients (ICCs) and Bland–Altman plots. *Results:* The MUAR at the carpal tunnel inlet showed a strong positive correlation with the MUR at the same level (*ρ* = 0.738, *p* < 0.05) and the median nerve wrist forearm ratio (*ρ* = 0.541, *p* < 0.05). In addition, the MUAR at the carpal tunnel outlet presented a strong positive correlation with MUR at the carpal tunnel inlet (*ρ* = 0.528, *p* < 0.05). The MUAR at 12 cm proximal to the distal wrist crease showed a strong positive correlation with the MUR at the corresponding level (*ρ* = 0.613, *p* < 0.05). Intra-rater and inter-rater reliability showed a high degree of agreement (ICCs > 0.90). *Conclusions:* This study demonstrates correlations between known CTS parameters and MUAR and suggests the possibility of MUAR as a reliable CTS diagnostic tool. Further research is recommended to validate these findings.

## 1. Introduction

Carpal tunnel syndrome (CTS) is the most common type of entrapment neuropathy. It has a prevalence of 4% in the general population [1,2]. Recent research demonstrates that CTS has evolved from a simple entrapment neuropathy to a condition extensively explored in terms of epidemiology, imaging-based diagnosis, and several managements [3]. Due to its advantages of being non-invasive and painless, ultrasonography has increasingly been used for the diagnosis of CTS [4,5]. Especially, through emerging diagnostic modalities with multiparametric ultrasound, CTS has been extensively investigated with integrating morphometric, vascular, and biomechanical parameters. This approach encompasses dynamic assessment, vascular imaging, and elastography (strain and shear-wave), therefore, it provides comprehensive insights into nerve stiffness and microcirculation. This enhances the diagnostic accuracy of CTS beyond simple cross-sectional area (CSA) measurement. Therefore, ultrasound is considered as a noninvasive and reproducible alternative to electrodiagnostic studies (EDX) [6,7,8]. 

Along with this research trend, recent systematic review and meta-analysis demonstrate that neuromuscular ultrasound (NMUS) achieves diagnostic accuracy comparable to EDX for CTS [9]. Furthermore, the direct association between electrodiagnostic severity and ultrasonographic measurements of the median nerve has led to a vigorous increase in the utilization of ultrasonography for CTS diagnosis [10]. The CTS ultrasonographic criteria have been studied in-depth using high-resolution NMUS [11,12,13]. 

In the ultrasonographic evaluation of CTS, several studies have identified the usefulness of the CSA ratio between the median and ulnar nerves in diagnosing CTS. The median-to-ulnar nerve cross-sectional area ratio (MUR) is a widely used ultrasonographic marker for the diagnosis of CTS. The MUR is considered an effective parameter because it is obtained simultaneously at the wrist level and correlates with the electrophysiological severity of CTS [14]. 

Despite these advantages, CTS status can affect the ulnar nerve owing to wrist anatomy and disease pathophysiology [15,16,17]. It can naturally lead to the possibility of decreased diagnostic value of MUR [18]. 

Therefore, we focused on the utility of the ulnar artery as a CTS diagnostic parameter. The ulnar artery travels superficially to the flexor retinaculum and laterally to the ulnar nerve. Because of this anatomical location, the ulnar artery is assumed to be less affected by CTS-related conditions. In addition, several studies have shown no correlation between the distal upper arm arterial diameter and body parameters such as body mass index (BMI), body surface area, height, and weight [19,20]. Therefore, we considered that the ulnar artery could be used as a stable control parameter that is not seriously affected by other factors. 

We calculated the ratios with the median nerves to establish the clinical significance of the ulnar artery as a robust parameter for the diagnosis of CTS using ultrasonography. We sought to identify whether the median nerve-to-ulnar artery cross-sectional area ratio (MUAR) is related to the existing CTS parameters.

This study aimed to demonstrate that the MUAR correlates with previously well-known ultrasonographic parameters used as diagnostic indicators for CTS.

## 2. Materials and Methods

### 2.1. Study Design

A retrospective study was conducted in Korea University Guro Hospital, Republic of Korea. This study was approved by the institutional review board (IRB) of Korea University Guro Hospital (2025GR0257). After obtaining IRB approval, we analyzed the clinical records of patients with CTS in our outpatient service and selected those with CTS confirmed using EDX. EDX was performed based on the criteria developed by the American Association of Neuromuscular and Electrodiagnostic Medicine guidelines [21].

From March 2021 to December 2024, the clinical records of 67 wrists with newly diagnosed CTS from 42 patients were obtained. The inclusion criteria were as follows: (1) age between 18 and 80 years, and (2) newly diagnosed CTS using EDX. The exclusion criteria were as follows: (1) history of surgery for CTS, (2) history of wrist fractures or other wrist surgeries, (3) diagnosis of other concurrent neurological problems (e.g., other peripheral mononeuropathy and/or polyneuropathy and/or radiculopathy), (4) poor cooperation, and (5) difficulty in maintaining posture for evaluation. 

The reviewed data were recorded in a structured format. We collected information of the numeric rating scale (NRS), Boston Carpal Tunnel Syndrome Questionnaire (BCTQ) symptom severity scale (BCTQ-SSS), and BCTQ functional status scale (BCTQ-FSS). In addition, the results of physical examinations related to CTS, such as Tinel’s sign, Phalen’s test, and abductor pollicis brevis (APB) muscle atrophy were included. The presence or absence of nocturnal numbness, a typical symptom of CTS, was also recorded.

### 2.2. Ultrasonographic Examination and Measurements

High-resolution NMUS evaluation was performed after the EDX examination using an RS80A ultrasound scanner (Samsung Medison Co. Ltd., Seoul, Republic of Korea). Evaluation was conducted using a linear array transducer operating at 3.0–12.0 MHz. This frequency range was selected to optimize spatial resolution for superficial musculoskeletal structures, including tendons, ligaments, and nerves, while maintaining adequate penetration for the wrist. 

The patients were placed in the supine position with their elbows and wrists in neutral positions. Ultrasound examinations were performed by one physical medicine and rehabilitation physician who trained in the musculoskeletal subspecialty. 

**Figure 1:**
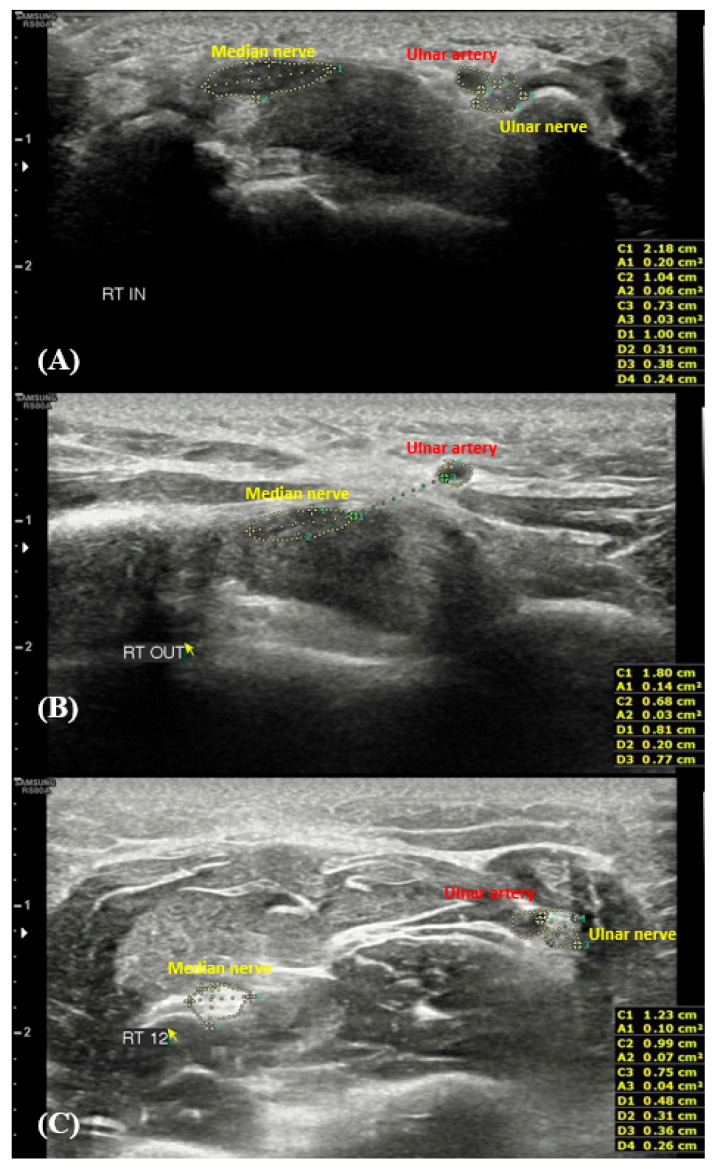
Transverse ultrasonography revealing the median nerve, ulnar nerve, and ulnar artery. The carpal tunnel inlet (**A**), carpal tunnel outlet (**B**), and 12 cm proximal to the distal wrist crease (**C**).

The examiner first detected the median nerve at the carpal tunnel inlet (level of the scaphoid-pisiform), then continuously traced along the inner border of the epineurium when evaluating the nerves. Thus, the examiner measured the median and ulnar nerves at the carpal tunnel inlet, carpal tunnel outlet (the level of the hook of the hamate), and 12 cm proximal to the distal wrist crease [22]. The ulnar artery was identified using power and color Doppler sonography. The transducer was applied with minimal pressure to prevent vessel compression and subsequent alteration of Doppler findings. After confirming the artery location and the inner border of the artery at maximal dilation, the ulnar artery CSA was captured. The ulnar artery CSA was measured at three predefined levels, the sites where the median and ulnar nerves were assessed. Transverse ultrasonographic views of the median nerve, ulnar nerve, and ulnar artery at the three levels are shown in Figure 1. 

Using these parameters in all three areas, we determined the median nerve CSA (mm^2^), median flattening ratio (MFR), MUR, and median nerve wrist forearm ratio (WFR). In addition, we measured MUAR as a new parameter by dividing the median nerve CSA by the ulnar artery CSA. We analyzed the diagnostic utility of MUAR by comparing it with other CTS parameters.

### 2.3. Statistical Analysis

For the descriptive analysis, the median and interquartile ranges of the measurements were used for the metric evaluations. Using Spearman’s correlation coefficient, we demonstrated that MUAR was correlated with other CTS parameters. To evaluate the reproducibility of the MUAR measurements, intraclass correlation coefficients (ICCs) were calculated. Intra-rater reliability was assessed by comparing two separate sets of measurements obtained by each reader, while inter-rater reliability was evaluated by comparing the measurements between the two readers. We interpreted ICC values as follows: <0.5, ‘poor’; 0.50–0.75, ‘moderate’; 0.75–0.90, ‘good’; and >0.90, ‘excellent’ [23]. In addition, Bland–Altman plots were constructed to analyze the agreement. The 95% limits of agreement were calculated as the mean difference plus and minus 1.96 of the standard deviation of the difference. SPSS (version 29.0; SPSS Inc., Chicago, IL, USA) and MedCalc (version 18.2.1; MedCalc Software Ltd., Ostend, Belgium) were used to perform all analyses, and <0.05 was considered statistically significant. 

## 3. Results

### 3.1. Characteristics and Clinical Data

The demographic characteristics and clinical data of the study participants are shown in Table 1. The participants comprised 13 males and 29 females. The median age of the participants was 56 (52–62) years and the median BMI was 23.7 (21.4–26.2). Additionally, 40 participants were right-handed, and 2 were left-handed. Of the patients with CTS, 10 had right-sided CTS and 7 had left-sided CTS. Among the CTS wrists, 19 had mild CTS, 33 had moderate CTS, and 15 had severe CTS. The median duration of disease was 6 (3–12) months. The median NRS was 4 (2–6), the median BCTQ-SSS was 27 (21–34), and the median BCTQ-FSS was 14 (9–19). Tinel’s sign was positive in 32 wrists and negative in 35 wrists. Phalen’s test results were positive in 39 wrists and negative in 28 wrists. APB atrophy was observed in 11 wrists and was negative in 56 wrists. Nocturnal numbness in digits I–III was present in 43 wrists and was absent in 24 wrists.

### 3.2. Ultrasonographic CTS Parameters

The ultrasonographic parameters of CTS, ulnar artery CSA, and MUAR measurements are shown in Table 2. At the carpal tunnel inlet, the median CSA (mm^2^) was 14.0 (11.0–16.5), MFR was 3.20 (2.82–3.56), MUR was 3.0 (2.06–4.29), ulnar artery CSA (mm^2^) was 4.0 (3.0–6.0), and MUAR was 3.20 (2.31–4.90). At the carpal tunnel outlet, the median CSA (mm^2^) was 10.2 (9.0–13.0), MFR was 3.26 (2.61–3.80), ulnar artery CSA (mm^2^) was 4.0 (3.0–5.0), MUAR was 2.75 (2.0–4.0), and median nerve WFR was 1.80 (1.56–2.53). At 12 cm proximal to the distal wrist crease, the median CSA (mm^2^) was 7.0 (6.0–9.0), MFR was 2.0 (1.61–2.31), MUR was 1.40 (1.0–1.69), ulnar artery CSA (mm^2^) was 5.0 (4.0–6.5), and MUAR was 1.50 (1.16–2.0). The parameters were either similar or had decreased between the carpal tunnel inlet and the outlet. In addition, when comparing the carpal tunnel inlet and 12 cm proximal to the distal wrist crease, all parameters decreased, except for the ulnar artery CSA. 

### 3.3. Correlation Between MUAR and Other CTS Parameters

The MUARs in the three areas were positively correlated. The MUAR at the carpal tunnel inlet demonstrated a strong positive correlation with that at the carpal tunnel outlet (Spearman’s rho = 0.747, *p* < 0.001). The MUAR at the carpal tunnel inlet was positively correlated with the MUAR at 12 cm proximal to the distal wrist crease (Spearman’s rho = 0.389, *p* = 0.001). In addition, the MUAR at the carpal tunnel outlet was positively correlated with the MUAR at 12 cm proximal to the distal wrist crease (Spearman’s rho = 0.344, *p* = 0.005). 

The correlation coefficients between the MUAR in each of the three areas and the other CTS parameters are presented in Table 3. The MUAR at the carpal tunnel inlet exhibited a strong positive correlation with the MUR at the same level and the median nerve WFR. The MUAR at the carpal tunnel inlet was positively correlated with the median nerve CSA at the same level and the MFR at the carpal tunnel outlet. In addition, the MUAR at the carpal tunnel outlet showed a strong positive correlation with MUR at the carpal tunnel inlet. The MUAR at the carpal tunnel outlet was positively correlated with the median nerve CSA at the carpal tunnel inlet, median nerve CSA, MFR at the same level, and the median nerve WFR. In addition, the MUAR at 12 cm proximal to the distal wrist crease showed a strong positive correlation with the MUR at the same level. The MUAR at 12 cm proximal to the distal wrist crease was positively correlated with the MUR at the carpal tunnel inlet and median nerve CSA at the same level.

Regarding the location of the measurements, the median nerve CSAs in the three areas suggested positive correlations with MUAR at the matched (same) levels. The MFR at the carpal tunnel outlet was positively correlated with the MUAR at the corresponding level. The MUR at the carpal tunnel inlet and 12 cm proximal to the distal wrist crease exhibited a strong positive correlation with MUAR at the matched levels. These significant positive correlations between MUAR and the parameters measured at different levels were confirmed but did not exhibit clear patterns.

**Figure 2:**
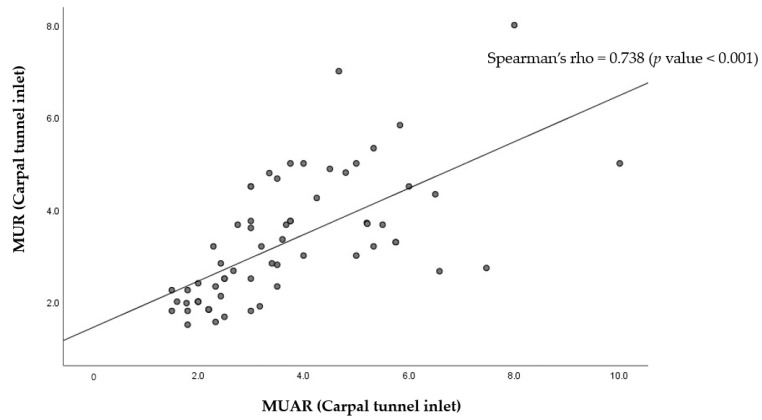
A scatter plot of the correlation between MUAR and known CTS diagnostic parameters. MUAR, median nerve-to-ulnar artery ratio; CTS, carpal tunnel syndrome.

A scatter plot representing the strong correlation between the CTS parameters and MUAR is shown in Figure 2. 

### 3.4. Intra-Rater and Inter-Rater Reliability

**Figure 3:**
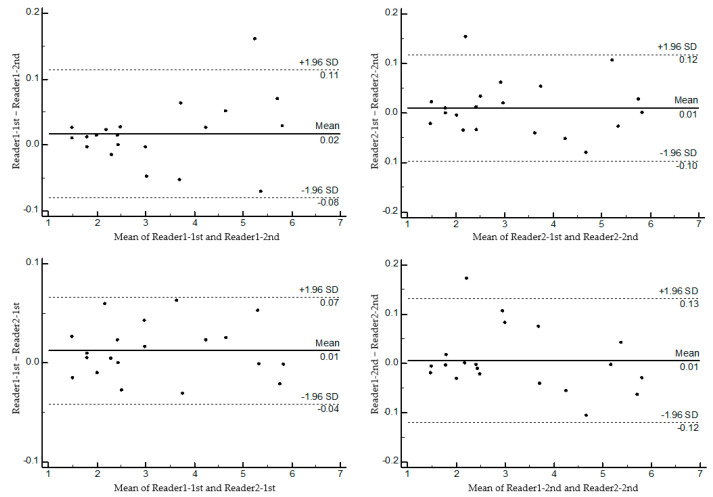
Bland–Altman plots of the agreement between readers’ MUAR measurements. The solid line represents the mean difference, and the dashed lines represent the 95% limits of agreement. SD, standard deviation.

The intra-rater and inter-rater reliabilities of two readers’ MUAR measurements demonstrated excellent agreement with an ICC of >0.90 with a 95% confidence interval. The agreement results are illustrated graphically on the Bland–Altman plots (Figure 3). They showed no evidence of systematic bias based on the degree of the mean area.

## 4. Discussion

Well-established ultrasonographic findings have been introduced and inspected for validation. One of the most representative parameters is the MUR; however, this is controversial because the MUR may be affected by the CTS. Recently, CTS has affected the ulnar nerve under various conditions. A previous study reported significant changes in the ulnar nerve in a nerve conduction study and ultrasonographic evaluation of CTS. They revealed that CTS status can electrophysiologically affect the ulnar nerve [15]. Another ultrasonographic study suggested that the CSAs of the ulnar and median nerves at the wrist are increased in patients with CTS, and that, after release of the transverse carpal ligament, the CSA of the ulnar nerve was significantly lower than that measured before the operation [17]. Similarly, the ulnar nerve in CTS and Guyon’s canal was correlated with the CTS release operation. It had a considerable effect not only on the anatomy of Guyon’s canal but also on the morphological and functional status of the ulnar nerve [16]. More specifically, there was one report on the reliability of ultrasonographic measurements in CTS and their correlations with symptom duration and electrophysiologic findings. Only pisiform CSA measurements were significantly diagnostic of early CTS, and the MUR had a weaker diagnostic value for CTS [18]. Due to these conflicts, there are issues surrounding the diagnostic reliability of ulnar nerve measurements for CTS.

Recently, multiparametric ultrasound has expanded the clinical and research scope of CTS. Especially, focused on vascular evaluation, this can be divided into conventional Doppler ultrasound techniques and superb microvascular imaging (SMI) [6]. Conventional Doppler ultrasound techniques including power and color Doppler, have been applied to evaluate intraneural vessels in CTS [24]. Power Doppler ultrasound grades vascularity in a valid and reliable method, and increased intraneural flow correlates with disease severity [25,26]. However, the sensitivity and specificity vary widely because of several factors, including disease severity [27]. Color Doppler ultrasound demonstrates more consistent diagnostic performance, particularly in moderate and severe CTS showing hyper-vascularization [28]. SMI has been used to visualize the low-velocity smaller intraneural vessels by suppressing motion artifacts without contrast medium [29]. Recent studies have applied vendor-based vascular index and manual vessel counts to quantify intraneural micro-vascularity. Vendor-based measurements showed poor correlation with manually calculated results, indicating that manual counting may be more reliable [30]. There is an additional necessary consideration about the frequency of transducers. Generally, high-frequency transducers (10–16 MHz) are used to detect intraneural vessels in CTS. Therefore, lower-frequency probes may fail to depict micro-vasculature, leading to potential false-negative findings [29,30,31].

Based on these recent research trends, we decided to shed light on a new approach for identifying stable ultrasonographic parameters that can be used for CTS diagnosis. We selected the ulnar artery for the measurements. No prior studies have included ulnar artery investigations in the diagnosis of CTS other than a Doppler evaluation of the radial and ulnar arteries in patients with CTS [32]. We first proposed the ulnar artery as a new ultrasonographic parameter for CTS. In addition to measuring the CSA of the artery, for better analysis, we considered it reasonable to calculate the ratio using the median nerve CSA. We obtained other CTS ultrasonographic parameters to determine the correlation between MUAR and these parameters. 

In a descriptive view, the measured parameters generally decreased from the carpal tunnel inlet to the outlet and from the carpal tunnel inlet to 12 cm proximal to the distal wrist crease. The MUAR at the carpal tunnel inlet, carpal tunnel outlet, and 12 cm proximal to the distal wrist crease were significantly correlated with each other. We considered that this was a result of the MUAR being measured at each level, which increased the likelihood of each other’s diagnostic value. MUAR at the carpal tunnel inlet and outlet demonstrated a strong correlation. This suggests that measurements at these two points may be important.

The key difference between MUAR and other CTS parameters may depend on the measurement level. Therefore, correlations involving the same measurement level should be considered. The median nerve CSA was positively correlated with the MUAR in all matched areas. In addition, the MFR at the carpal tunnel outlet was positively correlated with the MUAR at the same level. These results imply the possibility of using MUAR as a meaningful indicator for the diagnosis of CTS. The MUR at the carpal tunnel inlet appeared to have a strong positive correlation with the MUAR at the same levels. In addition, the correlation measured 12 cm proximal to the distal wrist crease showed a similar pattern. This suggests that MUAR may be an alternative to MUR as a diagnostic tool for CTS.

This study has several limitations. First, being retrospective in nature may have introduced selection and information biases. Second, findings were derived from a single-center cohort, which may cause errors in generalization to other populations. Third, because all measurements in this study were obtained by a single operator, this design may have introduced measurement bias and limited external reproducibility. Therefore, the generalizability of the measurement results should be interpreted with caution. Fourth, the timing of arterial measurement was determined by the operator’s subjective judgment rather than by a standardized protocol. Such operator-dependent decisions may have introduced observer or measurement bias, as the timing could have been influenced by the clinical condition or the operator’s perception of the subject’s status. Fifth, the measurement was performed with a transducer with relatively lower frequencies; it may have failed to depict micro-structures and caused potential sensitivity loss for CSA delineation and microvascular assessments. Sixth, as patients with moderate and severe CTS were more likely to be included in the analysis, our study may be subject to selection or referral bias and it potentially leads to difficulty in generalizing the results. Seventh, there was no control group in our study. As the study was intended to suggest new diagnostic criteria, there were limitations, and further research is needed to evaluate its validity as a diagnostic performance.

## 5. Conclusions

To the best of our knowledge, this is the first study focused on the ulnar artery in the diagnostic evaluation of CTS, which suggests the possibility of MUAR as an ultrasonographic parameter for the diagnosis of CTS. However, the present results do not directly validate its diagnostic accuracy. Further prospective, blinded, case–control, or diagnostic cohort study is needed. We expect that our study will have a meaningful impact on the value of ultrasonography in diagnosing CTS.

## Figures and Tables

**Table 1:** General demographic characteristics and clinical data of the study participants.

Characteristics and Clinical Data	
Patient number	42
Sex	Male: 13, Female: 29
Age (y)	56 (52–62)
BMI (kg/m^2^)	23.7 (21.4–26.2)
Dominant hand	Right: 40, Left: 2
Lesion side	Right: 10, Left: 7, Bilateral: 25
CTS wrist number	67
Severity	Mild: 19, Moderate: 33, Severe: 15
Duration (months)	6 (3–12)
NRS	4 (2–6)
BCTQ-SSS	27 (21–34)
BCTQ-FSS	14 (9–19)
Tinel’s sign	Positive: 32, Negative: 35
Phalen’s test	Positive: 39, Negative: 28
APB atrophy	Positive: 11, Negative: 56
Nocturnal numbness in digit I–III	Presence: 43, Absence: 24

Note: Values are reported as median and interquartile range. Abbreviations: BMI, body mass index; CTS, carpal tunnel syndrome; NRS, numeric rating scale; BCTQ, Boston Carpal Tunnel Syndrome Questionnaire; SSS, symptom severity scale; FSS, functional status scale; APB, abductor pollicis brevis.

**Table 2:** Comparisons of the ultrasonographic parameter values at the three levels analyzed.

Parameters	Carpal Tunnel Inlet	Carpal Tunnel Outlet	12 cm Proximal to the Distal Wrist Crease
Median nerve CSA (mm^2^)	14.0 (11.0–16.5)	10.2 (9.0–13.0)	7.0 (6.0–9.0)
MFR (long/short diameter)	3.20 (2.82–3.56)	3.26 (2.61–3.80)	2.0 (1.61–2.31)
MUR	3.0 (2.06–4.29)	*	1.40 (1.0–1.69)
Ulnar artery CSA (mm^2^)	4.0 (3.0–6.0)	4.0 (3.0–5.0)	5.0 (4.0–6.5)
MUAR	3.20 (2.31–4.90)	2.75 (2.0–4.0)	1.50 (1.16–2.0)
Median nerve WFR	1.80 (1.56–2.53)		

Note: Values are reported as the median and interquartile range. Abbreviations: CSA, cross-sectional area; MFR, median flattening ratio; MUR, median-to-ulnar nerve CSA ratio; MUAR, median nerve-to-ulnar artery ratio; WFR, wrist-to-forearm ratio. * The ulnar nerve was not evaluated because it branched at this level.

**Table 3:** Spearman’s correlation coefficient between CTS ultrasonographic parameters and MUAR at each level.

Parameters		MUAR at the Carpal Tunnel Inlet	MUAR at the Carpal Tunnel Outlet	MUAR at 12 cm Proximal to the Distal Wrist Crease
Carpal tunnel inlet	Median nerve CSA (mm^2^)	0.485 *	0.272 *	0.145
	MFR (long/short diameter)	0.219	0.206	0.237
	MUR	0.738 *	0.528 *	0.299 *
Carpal tunnel outlet	Median nerve CSA (mm^2^)	0.211	0.328 *	0.023
	MFR (long/short diameter)	0.325 *	0.287 *	0.213
	MUR	†	†	†
12 cm proximal to the distal wrist crease	Median nerve CSA (mm^2^)	–0.069	–0.137	0.424 *
	MFR (long/short diameter)	0.007	–0.046	–0.074
	MUR	0.165	0.192	0.613 *
Median nerve WFR		0.541 *	0.396 *	–0.231

Abbreviations: CSA, cross-sectional area; MFR, median flattening ratio; MUR, median-to-ulnar nerve CSA ratio; MUAR, median nerve-to-ulnar artery ratio; WFR, wrist-to-forearm ratio. † The ulnar nerve was not evaluated because it branched at this level. * *p* < 0.05.

## Data Availability

The data presented in this study are available on request from the corresponding author due to institutional policy and patient privacy regulations.

## Abbreviations

The following abbreviations are used in this manuscript:

APB	Abductor pollicis brevis
BCTQ	Boston carpal tunnel syndrome questionnaire
BMI	Body mass index
CSA	Cross-sectional area
CTS	Carpal tunnel syndrome
EDX	Electrodiagnostic studies
FSS	Functional status scale
ICCs	Intraclass correlation coefficients
MFR	Median flattening ratio
MUAR	Median nerve-to-ulnar artery cross-sectional area ratio
MUR	Median-to-ulnar nerve cross-sectional area ratio
NMUS	Neuromuscular ultrasonography
NRS	Numeric rating scale
SMI	Superb microvascular imaging
SSS	Symptom severity scale
WFR	Wrist forearm ratio
